# Chemical Composition and Acaricidal Activity of *Lantana camara* L. Essential Oils Against *Rhipicephalus microplus*

**DOI:** 10.3390/plants14152336

**Published:** 2025-07-29

**Authors:** Jorge Ramírez, Karla Balcázar, Jéssica López, Leydy Nathaly Castillo, Ruth Ortega, Haydee Vidal López, Ernesto Delgado-Fernández, Wilmer Vacacela, James Calva, Chabaco Armijos

**Affiliations:** 1 Departamento de Química, Universidad Técnica Particular de Loja, Loja 1101608, Ecuador; lncastillo@utpl.edu.ec (L.N.C.); jwcalva@utpl.edu.ec (J.C.); cparmijos@utpl.edu.ec (C.A.); 2 Carrera de Bioquímica y Farmacia, Universidad Técnica Particular de Loja (UTPL), Calle M. Champagnat s/n, Loja 1101608, Ecuador; kebalcazar1@utpl.edu.ec (K.B.); jelopez23@utpl.edu.ec (J.L.); 3 Carrera de Agronegocios, Unidad de Educación a Distancia y en Línea, Universidad Nacional de Loja, Loja 110103, Ecuador; ruth.ortega@unl.edu.ec; 4 Carrera de Medicina Veterinaria y Zootecnia, Universidad Nacional de Loja, Loja 110103, Ecuador; haydee.vidal@unl.edu.ec (H.V.L.); wilmer.vacacela@unl.edu.ec (W.V.); 5 INBIAM, Biotechnology and Environment Research Group, Universidad Politécnica Salesiana, Calle Vieja 12-30 y Elia Liut, Cuenca 010102, Ecuador; mdelgado@ups.edu.ec

**Keywords:** acaricidal efficacy, chemical composition, essential oil, GC-MS, *Lantana camara*, *Rhipicephalus microplus*

## Abstract

For the first time, essential oils (EOs) from the leaves and flowers of *Lantana camara* L., grown in Loja, Ecuador, have been isolated by steam distillation and analyzed. The oil yields from the extractions were 0.021 and 0.005% for the leaves and flowers, respectively. A compositional analysis using gas chromatography revealed the presence of EOs, comprising approximately 97.98% of the extract from the leaves and 74.58% of the extract from the flowers. The chemical characterization of these EOs indicated sesquiterpenic profiles. The most representative constituents of the essential oils from the flowers were γ-Curcumene (21.79%), (*E*, *E*)-α-Farnesene (20.07%), and α-Zingiberene (13.38%), while the EOs from the leaves were characterized by the abundant presence of γ-Curcumene (21.87%), (*E*)-Nerolidol (15.09%), and cis-Muurola-4(14),5-diene (12.65%). Furthermore, the acaricidal efficacy of the EOs from the leaves of *L. camara* was tested by a dip test with adult ticks, resulting in acaricidal efficacy at concentrations of 10%, demonstrating the useful properties of these EOs.

## 1. Introduction

The Verbenaceae family has 100 genera and approximately 2600 species, with the vast majority distributed in Latin America [1]. The best-known genera in this family are *Aloysia*, *Caryopteris*, *Citharexylum*, *Clerodendrum*, *Duranta*, *Lantana*, *Petrea*, *Stachytarpheta*, *Phyla*, *Verbena*, and *Vitex* [2,3]. *Lantana* has approximately 80 species distributed in tropical and subtropical America; these species are recognized for their ethnomedicinal, pharmaceutical, and ornamental uses [4]. *Lantana camara* is one of the most relevant species within this genus, commonly known in Ecuador as five blacks, holy blacks, seven colors, royal sage, supirosa, or venturosa [5]. This species, globally recognized as an ornamental plant, exhibits aromatic, small, and multicolored flowers. Its foliage consists of perennial, serrated, oval, and pubescent leaves. The plant typically ranges in height from 1 to 4 m and demonstrates a significant reproductive capacity, yielding up to 12,000 fruits annually [6,7]. *L. camara* has been used to treat and prevent pathologies, including cancer [8]. Its leaves are used for digestive and respiratory problems. Its root is used to purify the blood as well as for liver diseases [9]. In the same way, its infusion is used to treat tumors, measles, malaria and other health problems such as toothache, kidney issues, and digestive issues including diarrhea, vomiting, and flatulence. Other uses include treating burns, diabetes, pimples, and pangs [10,11,12]. 

Verbenaceae are well recognized for the presence of compounds, such as thymol, β-caryophyllene, citral, 1,8-cineole, carvona, and limonene, which are capable of modifying the permeability of the bacterial membrane, causing synergism with antibiotics and preventing the development of certain microorganisms without producing toxic effects [13]. Ecuador, recognized as a biodiverse nation, possesses a wealth of plant resources. However, despite this significant diversity, ethnomedicinal and phytotherapeutic research aimed at fully exploiting the properties of its flora remains limited. Consequently, there is a growing interest in conducting studies to identify the chemical composition of various native species [14].

Considering that essential oils (EOs) are the ideal prototype to be used as a raw material destined for different uses by the industry [15,16], and given that, until now, there have been no reports about the effects of *L. camara* derivatives on ticks, this work aimed to evaluate the acaricidal effects of the EOs obtained from *L. camara* against the common cattle tick *Rhipicephalus* (*Boophilus*) *microplus*. Taking into account that EOs are a promising source of naturally occurring bioactive compounds that show acaricide/insecticide activities, we hope that this work will be of interest to the scientific community in the field of natural products.

## 2. Results

### 2.1. Essential Oil Isolation

EOs of *L. camara* were obtained by steam distillation, with extraction yields of 0.021% (*v*/*w*) from the leaves and 0.005% (*v*/*w*) from the flowers.

### 2.2. Chemical Analysis of Essential Oils

**Figure 1:**
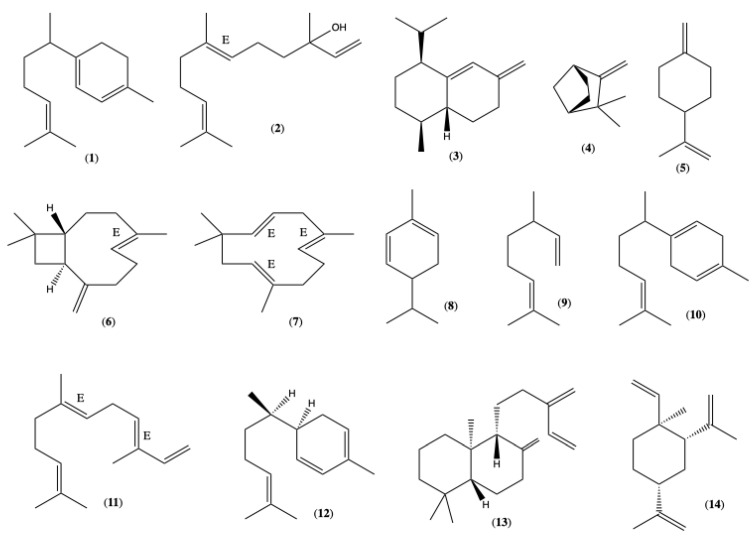
Chemical structures of the main components of the EOs of *L. camara* from Ecuador.

GC-MS and GC-FID analyses of *L. camara* EOs showed that hydrocarbon sesquiterpenes were the primary constituents of the chemical profile, accounting for 97.98% of the identified compounds of the leaf oils and 74.58% of the flower oils. As the principal components of leaves, the EOs are listed and shown in Figure 1: γ-Curcumene (**1**) (21.87 ± 0.10), (*E*)-Nerolidol (**2**) (15.09 ± 0.07), cis-Muurola-4(14),5-diene (**3**) (12.65 ± 0.20), Camphene (**4**) (5.63 ± 0.12), *p*-Mentha-1(7),8-diene (**5**) (4.91 ± 0.09), (*E*)-Caryophyllene (**6**) (4.29 ± 0.02), α-Humulene (**7**) (4.23 ± 0.02), α-Phellandrene (**8**) (3.69 ± 0.07), Myrcene (**9**) (2.93 ± 0.04), and β-Curcumene (**10**) (2.79 ± 0.02). In the flowers’ EOs, the more abundant components were γ-Curcumene (**1**) (21.79 ± 0.68), (*E*,*E*)-α-Farnesene (**11**) (20.07 ± 1.36), α-Zingiberene (**12**) (13.38 ± 0.33), β-Curcumene (**10**) (5.34 ± 0.20), α-Humulene (**7**) (5.26 ± 0.16), Sclarene (**13**) (3.65 ± 2.68), and β-Elemene (**14**) (2.99 ± 0.57). Both chemical profiles obtained in this study are detailed in Table 1, the gas chromatograms of EOs are available Supplementary Material.

### 2.3. Acaricidal Effect of L. camara Essential Oil

The highest value of tick mortality (100%) was achieved with 10% and 15% EO *L. Allmara*. All results are shown in Table 2 and Table 3, respectively.

Table 2 shows the percentages of mortality observed in three distinct groups of engorged ticks subjected to varying treatments. The results indicated that Treatment 1 did not have any impact on the tested groups, while Treatments 2 and 3 resulted in 100% mortality, showing that these essential oils have strong acaricidal properties.

In Table 3, we have included specific results about the number of repetitions, treatment, ticks exposed, number of ticks dead, and mortality percentages.

### 2.4. ANOVA Analysis

In order to evaluate whether a significant difference existed between the mean mortality rates of the treatments, an ANOVA analysis was performed; the results are displayed in Table 4. Comparisons were conducted to visualize the statistical significance of differences in the dependent variable between the treatment control group and the remaining groups employing 10% and 15% concentrations.

The results of the analysis of variance indicated a significant difference between the control treatment and the evaluated treatments; however, no statistically significant differences were found between the treatments themselves.

## 3. Discussion

The chemical composition of the essential oils obtained from leaves and flowers of *L. camara* was elucidated. Gas chromatography-mass spectrometry analysis of the essential oil identified sesquiterpene hydrocarbons, with γ-curcumene being the main compound in both compositions (leaves: 21.87 ± 0.10; flowers: 21.79 ± 0.68).

The primary constituents of the essential oil extracted from *L. camara* flowers have been reported in several studies undertaken across diverse geographical locations. For instance, a study in Saudi Arabia identified caryophyllene oxide (10.6%), β-caryophyllene (9.7%), spathulenol (8.6%), γ-cadinene (5.6%), and trans-β-farnesene (5.0%) as the major components [18]. In Bregbo, southeastern Côte d’Ivoire, where flowers were collected during two distinct periods, the predominant compounds were (*E*)-β-caryophyllene (ranging from 19.2% to 36.6%) and α-humulene (ranging from 8.5% to 19.9%) [19]. Analysis of *L. camara* flower essential oil from Nigeria revealed sabinene (21.5%), 1,8-cineole (12.6%), β-caryophyllene (13.4%), and α-humulene (5.8%) as the most abundant constituents [20], whereas an analysis from India determined that the essential oil was predominantly composed of β-caryophyllene (26.9%), bicyclogermacrene (12.5%), and cis-davanone (7.4%) [21].

On the other hand, studies on the leaf essential oil of *L. camara* have shown differing profiles. One investigation reported nerolidol (*E*)-isomer, (43.4%), γ-cadinene (7.6%), and α-humulene (4.9%) as the main components [22]. Subsequently, in 2012, an analysis identified 71 compounds in the leaves, with β-caryophyllene, caryophyllene oxide, and β-elemene being the three most prevalent compounds [23]. Guerrero and Pozo identified 19 compounds as part of the whole compositions, with γ-muurolene (22.23%), trans-caryophyllene (17.07%), α-humulene (12.61%), γ-elemene (9.93%), and bicyclogermacrene (6.22%) being the most abundant [24]. Variations in the chemical compositions across these investigations can be attributed to several factors, including geographic location, time of collection, environmental conditions, season, temperature, and humidity. These parameters can significantly influence the relative abundance of the identified compounds.

The acaricidal effect of *L. camara* essential oil was evaluated at concentrations of 10% and 15% against *Rhipicephalus* (*Boophilus*) *microplus*, resulting in 100% mortality of adult ticks. Other studies have also evaluated the effectiveness of *L. camara* EO against *Rhipicephalus* (*Boophilus*) *microplus* through immersion tests on adults, demonstrating its effectiveness at a concentration of 100 mg/mL, whereby it notably reduced reproductive capacity by 55.65% [25]. Similarly, the acaricidal properties and safety of several plant materials, such as *Ptaeroxylon obliquum*, *Aloe ferox*, *L. camara*, and *Tagetes minuta*, used by rural farmers to control ticks on cattle, were evaluated. *L. camara* extracts at a 40% concentration showed an average tick load reduction of 58%, while the other plant species evaluated did not yield effective results [26]. The effectiveness observed in the present study may be attributed to the association of different active ingredients, potentially indicating synergism between active substances that optimize the action on *R.* (*B.*) *microplus*. According to Bakkali et al. and Showler [27,28], the acaricidal effects of EOs are associated with their bioactive compounds, highlighting the effects of multiple compounds which may act via multiple mechanisms against ectoparasites. γ-Curcumene, the major compound present in the EOs, has already been reported to exhibit various activities, including larvicidal and tickicidal effects, as demonstrated by Guzmán et al. [29], where the obtained results suggest that this compound should be further studied as a promising acaricide against *R. microplus*.

The control of *Rhipicephalus microplus*, an ectoparasite affecting cattle production worldwide, remains a major challenge for the livestock industry. It is estimated that *Rhipicephalus* species affect more than 80% of the global cattle population, causing significant economic losses due to reduced milk and meat production, transmission of pathogens, and costs associated with tick control [29]. In countries like Brazil and Mexico, annual losses attributed to *R. microplus* infestations have been estimated al USD 3.24 billion and USD 573.6 million, respectively [30]. In Ecuador, the situation is similarly concerning, where the unregulated and excessive use of synthetic acaricides has led to environmental contamination, food safety issues, and the emergence of acaricide-resistant tick populations, as reported by local veterinarians [29]. These challenges highlight the urgent need for alternative, sustainable, and eco-friendly control methods.

Although commercial biological control products exist for the control of ticks, many of these present limitations, such as high toxicity to non-target organisms, inconsistent efficacy, or rapid development of resistance. In this context, plant-derived essential oils have gained attention due to their biodegradability, low mammalian toxicity, and multiple modes of action, which reduce the likelihood of resistance development. Essential oils, such as those extracted from *Lantana camara*, have shown promising acaricidal activity due to the presence of bioactive compounds like sesquiterpenes and monoterpenoids, which interfere with the nervous system of arthropods, causing neurotoxic effects, paralysis, and death [31]. These natural compounds also exhibit repellent properties, which can prevent tick attachment and feeding, making them effective tools for integrated pest management strategies [29].

Previous studies have shown that *Lantana camara* essential oils exhibit high toxicity against *Rhipicephalus microplus* larvae, achieving mortality rates exceeding 90% at concentrations of 20 mg/mL. Studies have demonstrated that the essential oils from its leaves and flowers exhibit potent acaricidal activity [31,32,33,34,35]. This oil not only affects larval stages but also inhibits egg laying and larval development, suggesting its ability to interfere with the tick’s life cycle [35]. Moreover, it has been shown to significantly reduce oviposition and egg hatching, reinforcing its potential as a biocontrol agent. These findings support the feasibility of conducting new bioassays with this essential oil, especially in regions such as southern Ecuador, where phytochemical variability could yield even more promising effects.

## 4. Materials and Methods

### 4.1. Materials and Chemical Reagents

Standard aliphatic hydrocarbons for the GC-FID calibration curve were obtained from Chem Service (Sigma-Aldrich, St. Louis, MO, USA), and helium was supplied from INDURA (Quito, Ecuador). Anhydrous sodium sulfate was purchased from Sigma-Aldrich (San Luis, MO, USA). Olive oil and 95% ethanol were bought in local supermarkets. All solvents and reagents used were of analytical grade and were employed without further purification.

### 4.2. Plant Material

The collection of *L. camara*, authorized by the Ministry of the Environment of Ecuador (MAE), N°001-IC-FLO-DBAP-VS-DRLZCH-MA, took place during the late flowering stage in the Yaguarcuna neighborhood, Loja, Ecuador (4°11′10.518″ S–79°59′48.8148″ W). Once the plant material had been collected, it was transported to the Bioproducts Plant of the Universidad Técnica Particular de Loja, where the fresh leaves and flowers were separated prior to steam distillation for essential oil extraction.

### 4.3. Distillation of the Volatile Fraction

A total of 9 kg of leaves and 4.5 kg of flowers were separately subjected to hydrodistillation using a stainless steel Clevenger-type stainless steel apparatus for 90 min at atmospheric pressure. After the distillation was complete, the essential oil was dried using anhydrous sodium sulfate and subsequently stored at −4 °C.

### 4.4. Qualitative and Quantitative Analysis of the Essential Oils

Chemical compositions of the volatile fraction were analyzed using gas chromatography coupled with mass spectrometry (GC-MS). A Thermo Fisher Scientific model Trace 1310 gas chromatograph (GC), equipped with a Thermo Scientific AI/AS 1300 autosampler and an ISQ7000 single quadrupole mass spectrometer controlled by Chromeleon 7.2 Chromatography Data System (CDS) software (Waltham, MA, USA), was employed. The mass spectrometer operated with electron ionization at 70 eV, scanning a mass range of 40–350 *m*/*z*. Helium was used as the carrier gas at a constant flow rate of 1.00 mL/min. A 1 µL sample was injected into a DB-5 ms capillary column (5% phenylmethylpolysiloxane, 30 m × 0.25 mm internal diameter, 0.25 μm film thickness). The oven temperature program started at 60 °C (held for 5 min), then increased to 200 °C at a rate of 2 °C per minute, and finally reached 250 °C at a rate of 15 °C per minute (held for 5 min). The ion source and quadrupole temperatures were maintained at 230 °C and 150 °C, respectively. Each sample was analyzed in triplicate. The amount of each volatile component was determined using gas chromatography coupled with a flame-ionization detector (GC-FID). The same analytical conditions and column as the GC-MS method were used, with a split ratio of 1:40.

Individual compounds were identified by comparing their mass spectra and linear retention indices (LRIs) based on data reported in scientific literature [17]. The LRIs were experimentally calculated using the method described by Van Den Dool and Kratz [36], by injecting a series of straight-chain alkanes (C9 to C24). The relative percentage of each identified compound was calculated based on the normalized peak area relative to the total area of all identified compounds in the chromatogram.

### 4.5. Evaluation of the Acaricidal Effect of the Essential Oil

The study population was 90 adult ticks of the genus *Rhipicephalus* (*Boophilus*) *microplus*, obtained from adult cattle from the Ceibopamba sector of the Malacatos parish of the Loja canton, which were randomly sampled. The ticks were then divided into three experimental groups with 10 observational units each, distributed for the three treatments.

#### 4.5.1. Dip Test of Adult Ticks

This study employed a modified engorged female immersion test, based on the protocol by Drummond et al. [37] and adapted by FAO [38], to evaluate ixodicide efficacy. Engorged adult female ticks (n = 90) were subjected to different concentrations of test substances for thirty minutes, followed by seven days of incubation at 27 °C and 80–90% relative humidity. The experimental setup utilized a humidity chamber, water, a 200 mL beaker, a glass stirring rod, markers for identification, nine Petri dishes, 24-well culture plates, an incubator with controlled temperature and humidity (verified by a thermohygrometer), and labeling tape. *L. camara* essential oil and olive oil served as the positive and negative controls, respectively.

The experimental procedure involved an immersion bioassay of engorged female *Rhipicephalus* (*Boophilus*) *microplus* ticks to assess the acaricidal activity of *Lantana camara* essential oil (EO). A total of 90 surface-sterilized ticks (using 0.05% sodium hypochlorite, followed by rinsing and drying) were randomly assigned to three treatment groups (n = 30 ticks per group; 10 ticks per replicate). The groups were as follows: Group 1, treated with olive oil (100%) as the negative control; Group 2, treated with a 10% dilution of *L. camara* EO in ethanol; and Group 3, treated with a 15% dilution of *L. camara* EO in ethanol. Pure (undiluted) essential oil was not tested due to its high viscosity and potential for inconsistent application, as observed in preliminary trials. Each group of ten ticks was immersed in 30 mL of the respective solution for 30 min. After immersion, ticks were carefully removed using a fine mesh strainer, air-dried, and incubated under controlled conditions (27 ± 1 °C, 80% relative humidity, and a 12:12 h light:dark photoperiod). Mortality was evaluated after seven days by visually determining the proportion of dead ticks in each group [39].

#### 4.5.2. Statistical Analysis

A completely randomized experimental design was employed to evaluate the effect of EO concentration on tick mortality across replicates, which allowed us to make a comparison between treatment and repetitions. Data were subjected to one-way analysis of variance (ANOVA) using SPSS Statistics version 29.0, and the differences were considered statistically significant at *p* < 0.05.

## 5. Conclusions

Briefly, chemical analysis of *L. camara* essential oil from both the leaves and flowers revealed γ-Curcumene as the predominant sesquiterpene hydrocarbon. While this finding aligns with some reports on *L. camara* essential oil composition, significant variations exist across geographical locations, highlighting the influence of environmental factors and collection parameters on the chemical profile of essential oils. Notably, *L. camara* EO and its extracts have demonstrated promising acaricidal activity against the prevalent cattle tick *Rhipicephalus* (*Boophilus*) *microplus*, achieving high mortality rates and reducing reproductive capacity. Given the substantial global economic impact of *Rhipicephalus* infestations and the limitations of conventional control methods, the demonstrated efficacy of *L. camara* EO, potentially attributed to the synergistic action of its bioactive compounds like γ-Curcumene, warrants further investigation through comprehensive bioassays with a multi-target mechanism. While its effect may be slower, its eco-friendly profile and potential for developing sustainable formulations make it a promising alternative, especially for regions with high resistance to synthetic compounds.

Furthermore, the results of the present work contribute to the growing body of scientific evidence about the biological activity in *L. camara* and their products, as reported recently [40].

## Figures and Tables

**Table 1:** Chemical composition of the essential oils from leaves and flowers of *Lantana camara* L.

No.	Compound	LRI ^a^	LRI ^b^	Leaves	Flowers
				% ± SD	% ± SD
1	Sabinene	961	969	0.19 ± 0.00	-
2	Camphene	966	946	5.63 ± 0.12	-
3	*δ*-2-Carene	977	1001	0.16 ± 0.00	-
4	Verbene	980	961	0.03 ± 0.00	-
5	*α*-Phellandrene	993	1002	3.69 ± 0.07	0.12 ± 0.01
6	*p*-Mentha-1(7),8-diene	997	1003	4.91 ± 0.09	0.35 ± 0.03
7	Pentyl propanoate	1001	1005	1.28 ± 0.02	-
8	Myrcene	1005	988	2.93 ± 0.04	-
9	3-Octanol	1015	988	0.11 ± 0.00	-
11	*α*-Terpinene	1028	1014	0.10 ± 0.00	-
12	*ο*-Cymene	1032	1022	0.05 ± 0.00	-
13	Sylvestrene	1038	1025	1.12 ± 0.02	0.21 ± 0.02
14	1,8-Cineole	1042	1026	0.84 ± 0.01	-
15	(*Z*)-*β*-Ocimene	1045	1032	0.04 ± 0.00	-
16	(*E*)-*β*-Ocimene	1053	1044	0.51 ± 0.01	-
17	γ-Terpinene	1064	1054	0.33 ± 0.00	0.56 ± 0.02
18	cis-Sabinene hydrate	1078	1065	0.06 ± 0.05	-
19	Terpinolene	1089	1086	0.14 ± 0.11	-
20	Linalool	1106	1095	0.54 ± 0.01	-
21	n-Nonanal	1113	1100	0.07 ± 0.00	-
22	α-Fenchocamphorone	1116	1104	0.05 ± 0.00	-
23	trans-Pinocarveol	1146	1135	0.06 ± 0.00	-
24	cis-Verbenol	1148	1137	0.12 ± 0.00	-
25	trans-Verbenol	1153	1140	0.24 ± 0.00	-
26	Borneol	1179	1165	0.07 ± 0.01	-
27	Terpinen-4-ol	1186	1174	0.24 ± 0.01	-
28	α-Terpineol	1203	1186	0.30 ± 0.00	-
29	(3*Z*)-Hexenyl 3-methyl butanoate	1236	1232	0.08 ± 0.00	-
30	(2*Z*)-Hexenyl isovalerate	1242	1241	0.09 ± 0.00	-
31	Methyl citronellate	1249	1257	0.06 ± 0.00	-
32	Geranial	1279	1264	0.04 ± 0.00	-
33	trans-Pinocarvyl acetate	1300	1298	0.14 ± 0.00	-
34	δ-Elemene	1335	1335	0.23 ± 0.01	0.66 ± 0.23
35	α-Terpinyl acetate	1353	1346	0.08 ± 0.00	-
36	Eugenol	1364	1356	0.07 ± 0.00	-
37	Cyclosativene	1369	1369	0.03 ± 0.00	-
38	α-Copaene	1375	1374	0.28 ± 0.00	0.63 ± 0.28
39	β-Bourbonene	1383	1387	0.20 ± 0.01	0.33 ± 0.03
40	β-Cubebene	1388	1387	0.27 ± 0.00	-
41	β-Elemene	1390	1389	1.86 ± 0.12	2.99 ± 0.57
42	α-Funebrene	1402	1402	0.57 ± 0.00	0.97 ± 0.03
43	Italicene	1405	1405	0.01 ± 0.00	0.05 ± 0.02
44	α-Cedrene	1416	1410	0.28 ± 0.00	0.47 ± 0.01
45	(*E*)-Caryophyllene	1419	1427	4.29 ± 0.02	-
46	β-Ylangene	1424	1419	0.05 ± 0.00	-
47	cis-Thujopsene	1428	1429	0.39 ± 0.00	0.66 ± 0.02
48	β-Copaene	1431	1430	0.77 ± 0.03	-
49	β-Gurjunene	1431	1431	-	1.25 ± 0.18
50	α-trans-Bergamotene	1434	1432	0.03 ± 0.00	-
51	Aromadendrene	1439	1439	0.06 ± 0.00	0.55 ± 0.04
52	2-epi-*β*-Funebrene	1443	1411	0.32 ± 0.01	-
53	transMuurola-3,5-diene	1448	1451	0.03 ± 0.00	-
54	cis-Cadina-1(6),4-diene	1450	1461	0.10 ± 0.01	-
55	α-Humulene	1457	1452	4.23 ± 0.02	5.26 ± 0.16
56	Amorpha-4,11-diene	1459	1449	0.30 ± 0.01	-
57	9-epi-(E)-Caryophyllene	1461	1464	0.13 ± 0.01	-
58	α-Acoradiene	1468	1464	0.07 ± 0.00	0.01 ± 0.05
59	α-Neocallitropsene	1470	1474	0.20 ± 0.00	-
60	Dauca-5,8-diene	1474	1471	-	0.46 ± 0.05
61	γ-Muurolene	1475	1478	0.18 ± 0.00	-
62	cis-Muurola-4(14),5-diene	1484	1465	12.65 ± 0.20	-
63	ar-Curcumene	1485	1479	1.28 ± 0.02	-
64	Viridiflorene	1494	1496	0.10 ± 0.00	-
65	α-Zingiberene	1497	1493	-	13.38 ± 0.33
66	γ-Curcumene	1498	1481	21.87 ± 0.10	21.79 ± 0.68
67	α-Muurolene	1502	1500	0.06 ± 0.00	trace
68	cis-β-Guaiene	1506	1492	0.43 ± 0.06	-
69	β-Macrocarpene	1510	1499	-	0.47 ± 0.19
70	β-Curcumene	1513	1514	2.79 ± 0.02	5.34 ± 0.20
71	δ-Amorphene	1517	1511	0.41 ± 0.01	-
72	Cubebol	1521	1514	0.23 ± 0.00	-
73	δ-Cadinene	1522	1522	0.27 ± 0.00	0.63 ± 0.02
74	β-Sesquiphellandrene	1428	1421	0.04 ± 0.00	0.50 ± 0.02
75	trans-Calamenene	1536	1521	0.05 ± 0.00	-
76	Italicene ether	1540	1536	0.01 ± 0.00	-
77	Silphiperfol-5-en-3-ol A	1550	1557	0.02 ± 0.00	-
78	γ-Cuprenene	1559	1532	-	0.12 ± 0.01
79	trans-Dauca-4(11),7-diene	1558	1556	-	0.16 ± 0.01
80	trans-Sesquisabinene hydrate	1561	1577	0.09 ± 0.00	-
81	Germacrene B	1563	1559	1.01 ± 0.02	1.56 ± 0.07
82	(*E*,*E*)-α-Farnesene	1567	1505	-	20.07 ± 1.36
83	(*E*)-Nerolidol	1568	1561	15.09 ± 0.07	-
84	Spathulenol	1585	1577	0.70 ± 0.01	-
85	Caryophyllene oxide	1589	1582	0.14 ± 0.00	-
86	n-Hexyl benzoate	1589	1579	0.24 ± 0.03	-
87	Guaiol	1596	1600	0.09 ± 0.00	-
88	Viridiflorol	1601	1592	0.20 ± 0.01	-
89	Junenol	1613	1618	0.05 ± 0.00	-
90	Rosifoliol	1616	1600	0.14 ± 0.04	-
91	epi-Cedrol	1619	1618	-	0.29 ± 0.02
92	cis-Cadin-4-en-7-ol	1623	1635	-	1.62 ± 0.09
93	α-Corocalene	1628	1622	0.04 ± 0.00	-
94	Eremoligenol	1631	1629	0.07 ± 0.00	-
95	epi-α-Cadinol	1635	1638	0.24 ± 0.01	0.11 ± 0.01
96	β-Acorenol	1642	1636	0.18 ± 0.13	-
97	Himachalol	1642	1652	-	0.83 ± 0.05
98	1-epi-Cubenol	1644	1627	0.10 ± 0.14	-
99	Valerianol	1654	1656	-	0.19 ± 0.06
100	Cubenol	1654	1645	0.06 ± 0.00	-
101	epi-α-Muurolol	1656	1640	0.03 ± 0.00	-
102	α-Muurolol (Torreyol)	1659	1644	0.07 ± 0.01	0.45 ± 0.02
103	7-epi-α-Eudesmol	1659	1662	-	0.15 ± 0.04
104	α-Cadinol	1668	1652	0.08 ± 0.01	-
105	(*Z*)-α-Santalol	1680	1674	-	0.31 ± 0.04
106	β-Bisabolol	1680	1674	0.06 ± 0.01	-
107	11-αH-Himachal-4-en-1-β-ol	1695	1699	0.06 ± 0.01	-
108	Sclarene	1977	1974	-	3.65 ± 2.68
109	(6*E*,10*Z*)-Pseudo phytol	2034	2018	-	0.43 ± 0.14
	Monoterpene hydrocarbons			19.83	1.24
	Oxygenated monoterpenoids			2.58	-
	Sesquiterpene hydrocarbons			58.34	65.09
	Oxygenated sesquiterpenoids			15.27	3.94
	Others			1.96	4.31
	TOTAL			97.98	74.58

LRI ^a^, linear retention index calculated; LRI ^b^, linear retention index from [17]; %, percentage; SD, standard deviation. Both values were conveyed as means of three determinations.

**Table 2:** Mortality percentage of *Rhipicephalus* (*Boophilus*) *microplus* following treatment with different concentrations of *Lantana camara* essential oil, with olive oil as a control.

Engorged Ticks Group	Treatments		
	Olive Oil (1)	10% of *L. camara* EO (2)	15% of *L. camara* EO (3)
1	0	100	100
2	0	100	100
3	0	100	100

**Table 3:** Mortality percentages of *Rhipicephalus* (*Boophilus*) *microplus*.

Repetition	Treatment	No. of Ticks Exposed	No. of Dead Ticks	Mortality (%)
1	Olive oil (control)	10	0	0
1	10% of *L. camara* EO	10	10	100
1	15% of *L. camara* EO	10	10	100
2	Olive oil (control)	10	0	0
2	10% of *L. camara* EO	10	10	100
2	15% of *L. camara* EO	10	10	100
3	Olive oil (control)	10	0	0
3	10% of *L. camara* EO	10	10	100
3	15% of *L. camara* EO	10	10	100

**Table 4:** Analysis of variance (ANOVA) of mortality percentages in *Rhipicephalus* (*Boophilus*) *microplus* according to treatment.

S.V	S.S	df	MS	F	*p*-Value
Model	20,000.00	2	10,000.00	1.61 × 10^16^	<0.001
TTO	20,000.00	2	10,000.00	sd	sd
Error	3.7 × 10^−12^	6	0.00		
Total	20,000.00	8			

S.V = Source of variation, S.S = sum of squares, df = degrees of freedom, MS = mean square, F = F-statistic/F-ratio, *p*-value = probability value, Model = Statistical model, TTO = Treatment; error = Experimental error.

## Data Availability

The original contributions presented in this study are included in the article. Further inquiries can be directed to the corresponding authors.

## Supplementary Materials

The following supporting information can be downloaded at: https://www.mdpi.com/article/10.3390/plants14152336/s1.
