# Supplementary material for: Chemical Composition and Acaricidal Activity of Lantana camara L. Essential Oils Against Rhipicephalus microplus
Source: Plants (Basel). 2025 Jul 29;14(15):2336. doi: 10.3390/plants14152336 (PMC12348604; doi:10.3390/plants14152336)

Figure S.1

Gas chromatogram of *L. camara* flowers essential oil.

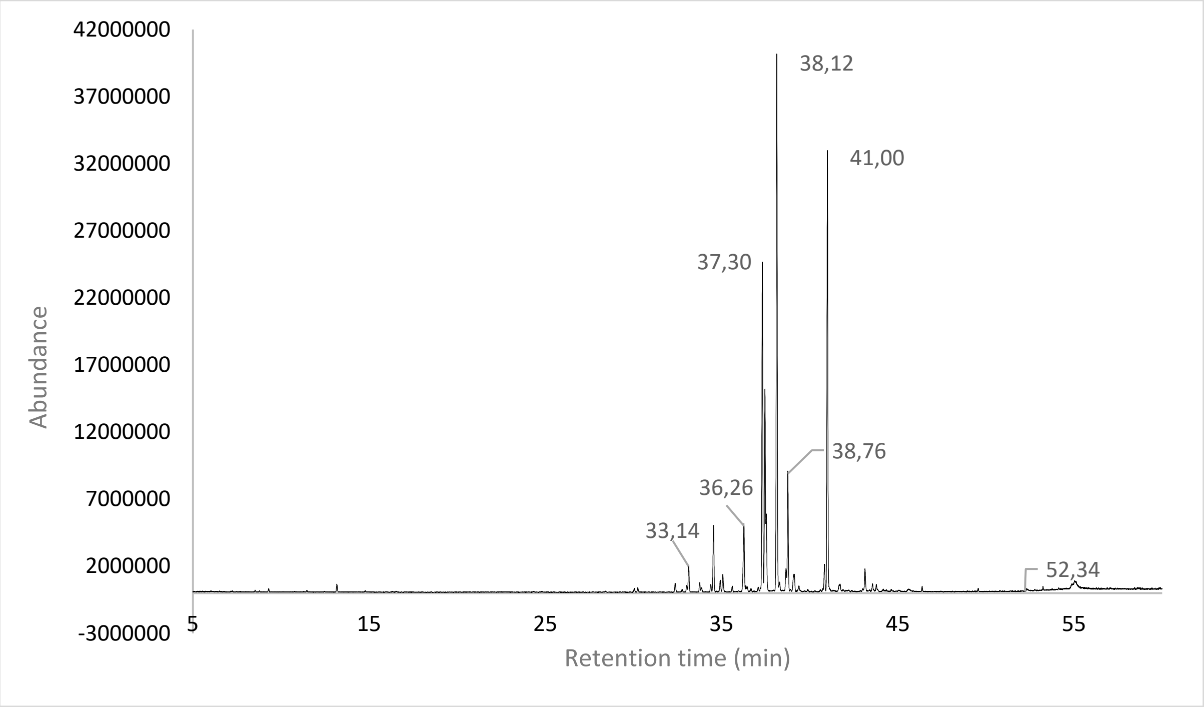

Figure S.2

Gas chromatogram of *L. camara* leaves essential oil.

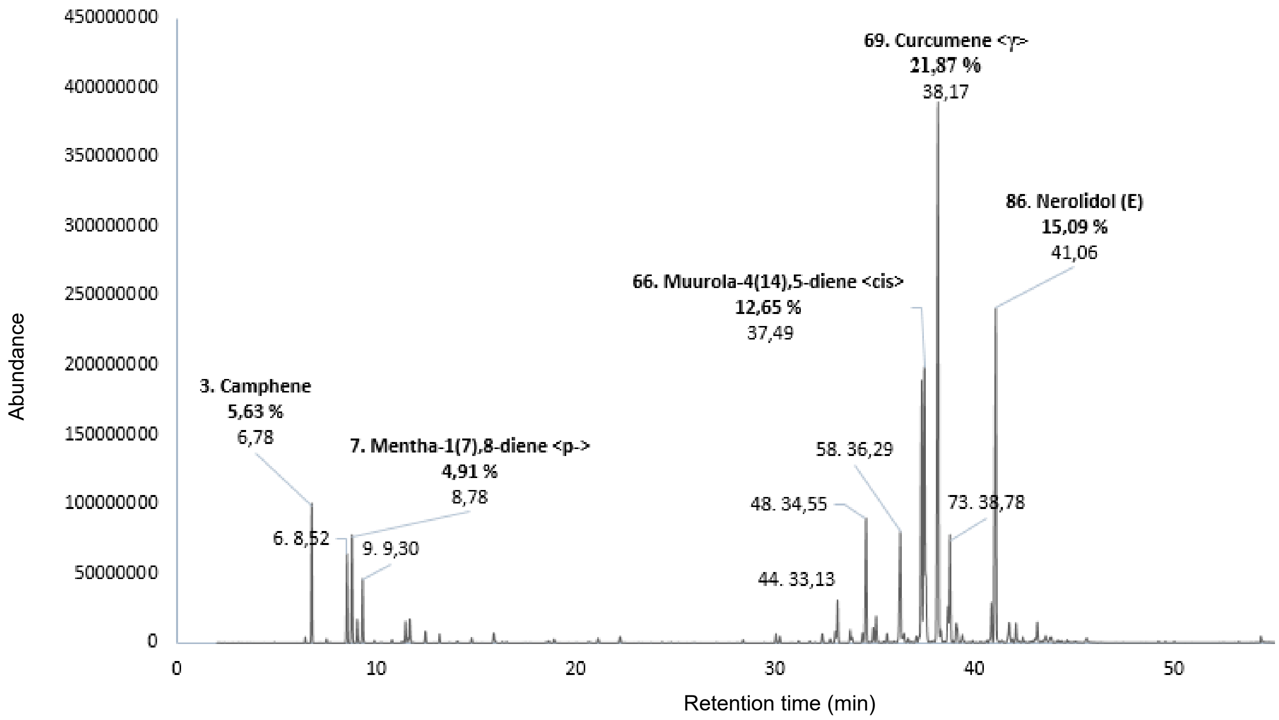

Supplement: Supplementary file 1 [file plants-14-02336-s001.zip › plants-3765465-supplementary.pdf]
